# Cystic Fibrosis Transmembrane Conductance Regulator (CFTR) Ubiquitylation as a Novel Pharmaceutical Target for Cystic Fibrosis

**DOI:** 10.3390/ph13040075

**Published:** 2020-04-22

**Authors:** Ryosuke Fukuda, Tsukasa Okiyoneda

**Affiliations:** Department of Biomedical Chemistry, School of Science and Technology, Kwansei Gakuin University, 2-1 Gakuen, Sanda, Hyogo 669-1337, Japan; r.fukuda@kwansei.ac.jp

**Keywords:** cystic fibrosis transmembrane conductance regulator (CFTR), endoplasmic reticulum quality control (ERQC), plasma membrane quality control (PMQC), ubiquitylation, chaperone, cystic fibrosis (CF) treatment

## Abstract

Mutations in the cystic fibrosis transmembrane conductance regulator (*CFTR*) gene decrease the structural stability and function of the CFTR protein, resulting in cystic fibrosis. Recently, the effect of CFTR-targeting combination therapy has dramatically increased, and it is expected that add-on drugs that modulate the CFTR surrounding environment will further enhance their effectiveness. Various interacting proteins have been implicated in the structural stability of CFTR and, among them, molecules involved in CFTR ubiquitylation are promising therapeutic targets as regulators of CFTR degradation. This review focuses on the ubiquitylation mechanism that contributes to the stability of mutant CFTR at the endoplasmic reticulum (ER) and post-ER compartments and discusses the possibility as a pharmacological target for cystic fibrosis (CF).

## 1. Introduction

Cystic Fibrosis (CF) is a lethal recessive disease caused by loss-of-function mutations in the *CFTR* (cystic fibrosis transmembrane conductance regulator). CF frequently occurs among Caucasians (about 1 in 3500), and the number of patients is more than 90,000 world-wide (US: approximately 30,000, Europe: approximately 48,000) [1]. CF patients present with progressive disease throughout the body, including in the respiratory tract, digestive tract, and exocrine organs (pancreas, testis, sweat glands, etc.). Shortly after birth, meconium ileus is caused by decreased intestinal lumen water volume and decreased secretion of digestive enzymes from the pancreas due to chronic pancreatitis. Found in the respiratory tract, bacterial infections such as *Pseudomonas aeruginosa* persist because airway clearance decreases due to an increase in the consistency of airway mucus. Chronic inflammation due to the infection promotes airway remodeling and fibrosis, gradually reducing respiratory function and leading to fatal respiratory failure. The life expectancy of CF patients has been prolonged due to the development of symptomatic treatments such as antibiotics, pancreatic digestive enzymes, expectorants, and anti-inflammatory drugs, but the median life expectancy is still about 40 years.

Cystic fibrosis transmembrane conductance regulator (CFTR) is a cyclic AMP (cAMP)-dependent chloride (Cl^−^) and HCO_3_^−^ ion channel that dominantly expresses at the apical plasma membrane (PM) of epithelial cells. CFTR is composed of two membrane-spanning domains (MSD1,2), a regulatory domain (R), and two nucleotide-binding domains (NBD1,2). More than 2000 CFTR mutations have been identified and classified as class I to class VII, based on their phenotypes [2]. The most prevalent CFTR mutation, ∆F508–CFTR, destabilizes NBD1 and the NBD–MSD domain–domain interaction, resulting in misfolding of the CFTR protein. ∆F508–CFTR is categorized as Class II, based on the instability at the endoplasmic reticulum (ER). Class II mutations cause abnormal CFTR protein folding at the ER and are recognized as misfolded proteins by the ER quality control (ERQC) system and, consequently, degraded by ER-associated degradation (ERAD). PM-localized rescued ∆F508 (r∆F508)–CFTR by correctors, or low-temperature rescuing, shows Class III (declined channel activity) and Class VI phenotypes. Class VI mutations do not compromise the trafficking to the PM expression and channel activity of the CFTR protein but cause rapid turnover of the CFTR protein at the PM due to accelerated endocytosis and lysosomal degradation by the PM quality control (PMQC) system. Because ubiquitylation levels determine the CFTR degradation both at the ER and PM [3,4], understanding the mechanism of CFTR ubiquitylation serves us the novel therapeutic strategy that could contribute to stabilizing the CFTR and improve the limited efficacy of CF drugs.

## 2. Biosynthesis of Cystic Fibrosis Transmembrane Conductance Regulator (CFTR) Protein

**Figure 1:**
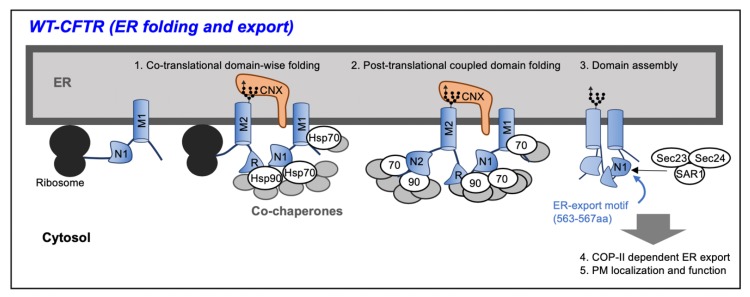
Wild type (WT)–cystic fibrosis transmembrane conductance regulator (CFTR) is translated at the ribosome on the endoplasmic reticulum (ER), and the membrane spanning domain (MSD) penetrates the ER membrane. Upon translation, each domain of the CFTR is folded with the assistance of chaperone–cochaperone complexes on the cytoplasmic side and inside of the ER to form a metastable structure (Co-translational folding). After the translation is completed, the domains interact by the action of the chaperones to form a native structure (Post-translational folding). Found in the nucleotide binding domain 1 (NBD1) of WT–CFTR, the ER export motif is exposed and CFTR is transported from the ER to the Golgi apparatus by coat protein complex II (COP II) vesicles.

CFTR is translated on the endoplasmic reticulum (ER) membrane and folds into the native conformation (Figure 1). The CFTR folding mechanism consists of the first step in which each domain folds into a metastable state during translation (co-translational domain-wise folding), and the second step in which each domain interacts after translation and folds into the native state as a whole CFTR protein (post-translational coupled-domain folding and assembly) [5,6,7,8]. CFTR undergoes N-glycosylation in the ER and interacts with calnexin (CNX), an ER lectin-like chaperone [9,10]. Cytoplasmic molecular chaperones Hsp70 and Hsp90, and their co-chaperones (Hdj2, p23, FKBP8), interact with the CFTR and assist its folding [11,12,13]. The most common ∆F508 mutation in cystic fibrosis (CF) is a mutation in which the 508th phenylalanine (F508) present in NBD1 is deleted, reducing the structural stability of NBD1 [14]. Since F508 is located on the surface of NBD1 and at the boundary between the NBD1 and intracellular loops of MSD1 and MSD2, it is thought that the ∆F508 mutation also destabilizes the interdomain interaction between NBD1 and MSD1 and MSD2 [7,15]. Translation kinetics also affects CFTR folding efficiency [16]. Ribosomal protein RPL12 knockdown has been reported to reduce the translation rate of CFTR mutants, resulting in partial improvement in ∆F508–CFTR folding [17]. Slowing of nascent polypeptide elongation with low concentrations of cycloheximide or emetine has been observed to improve the folding of ∆F508–CFTR [17].

## 3. Cystic Fibrosis Transmembrane Conductance Regulator (CFTR) Endoplasmic Reticulum Quality Control (ERQC)

**Figure 2:**
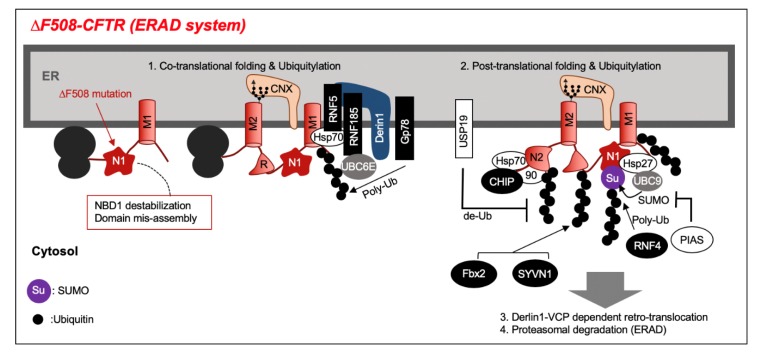
The stability of the interaction between nucleotide binding domain 1(NBD1) and membrane spanning domains (MSD1,2) decreases due to the NBD1 structural abnormality in the ∆F508–cystic fibrosis transmembrane conductance regulator (CFTR) mutation. The CFTR-associated E3 ligases recognize the misfolded regions mediated by chaperones and facilitate the co-translational and post-translational ubiquitylation. The endoplasmic reticulum (ER) export motif is folded inside ∆F508–CFTR and, conversely, it stays in the ER due to the exposure of the ER retention motif. Ubiquitylated ∆F508–CFTR is ultimately degraded by proteasome via retrotranslocation to the cytoplasm by the Derlin1– valosin-containing protein(VCP) complex.

Misfolded ∆F508–CFTR is retained in the endoplasmic reticulum (ER) by the ERQC system. CFTR export from the ER to the Golgi apparatus requires the CFTR di-acidic ER export motif (563–567aa, YKDAD) recognition by the Sec23/24 complex. CFTR misfolding veils this ER export motif and results in CFTR ER retention [18]. Simultaneously, CFTR misfolding exposes RXR-based ER retention/retrieval motifs and promotes CFTR ER retention [19,20]. ER retention of misfolded CFTR sustains interaction with cytosolic molecular chaperones Hsc70, Hsp70, and CNX [10,21,22,23]. ER retained misfolded CFTR will be ubiquitylated and retro-translocated to the cytoplasm by the Derlin-1 complex and degraded by the proteasome (Figure 2) [3,24,25,26].

Numerous ubiquitin ligases are involved in misfolded CFTR ubiquitylation at the ER (Figure 2). CFTR is ubiquitylated by co-translational and post-translational mechanisms [3,27]. An ER membrane-binding Really Interesting New Gene (RING)-type ubiquitin ligase Ring finger protein 5 (RNF5, also known as RMA1) recognizes the membrane-spanning region of CFTR and is involved in CFTR co-translational ubiquitylation [28]. An RNF5 homolog, Ring finger protein 185 (RNF185), localized at the ER membrane, also interacts with Derlin-1, and is involved in the CFTR co-translational ubiquitylation [29]. An ER membrane-localized RING-type ubiquitin ligase Gp78 (AMFR) has an E4-like activity and extends the CFTR ubiquitin chain produced by RNF5 [30]. A cytosolic chaperone binding ubiquitin ligase C-terminal Hsp-interacting protein (CHIP) recognizes the cytosolic region of CFTR and, especially, is involved in the post-translational ubiquitylation of ∆F508 CFTR–NBD2 [26,28]. The involvement of other ubiquitin ligases (Skp-Cullin-F box (SCF^Fbx2^), synoviolin 1 (SYVN1)) has been reported, but it is not clear if these ubiquitin ligases selectively recognize misfolded ∆F508 CFTR [31,32]. CFTR ubiquitylation at the ER is reversible, and USP19, an ER membrane tail-anchored de-ubiquitylating enzyme (DUB), has been reported to suppress the CFTR ER-associated degradation (ERAD) [33].

CFTR is not only ubiquitylated, but also modified by ubiquitin-like protein SUMO (SUMOylation). Hsp27, a small heat shock protein (sHsp), selectively recognizes ∆F508 CFTR and induces SUMOylation to cooperate with the SUMO E2 enzyme Ubc9. SUMOylated CFTR is ubiquitylated by RNF4, a SUMO-targeted Ub E3 ligase, and undergoes proteasome degradation [34]. Additionally, the Hsp27–Ubc9 complex selectively adds SUMO–2 to Lys447 of unfolded ∆F508–NBD1 [35]. SUMO E3 PIAS (protein inhibitor of activated STAT4) promotes SUMO–1 modification of CFTR and inhibits SUMO–2/3 modification, thereby inhibiting ubiquitylation of CFTR and suppressing its degradation [36].

## 4. Cystic Fibrosis Transmembrane Conductance Regulator (CFTR) Plasma Membrane Quality Control (PMQC)

Most of ∆F508–CFTR is eliminated by ER-associated degradation (ERAD), but some ∆F508–CFTR can escape from the endoplasmic reticulum quality control (ERQC) and reach the plasma membrane (PM). PM expression of ∆F508–CFTR has been observed in the airway and intestinal epithelial cells of cystic fibrosis (CF) patients [37]. ∆F508–CFTR folding is temperature-sensitive, and a low-temperature culture (26–30 °C) promotes the functional ∆F508–CFTR PM expression [38]. The CFTR corrector (lumacaftor, etc.), which is the main component of the CF drug Orkambi^®^, also promotes the PM expression of ∆F508–CFTR [39]. ∆F508–CFTR that appear on the PM can function as an ion channel but is ubiquitylated as an abnormal protein and rapidly removed from the PM [40,41,42]. Class VI CFTR mutants, such as ∆70–CFTR, are normally matured and transported to the PM but undergo ubiquitylation and rapid elimination from the PM [40]. Ubiquitylation promotes CFTR endocytosis and inhibits recycling. Ubiquitylated CFTR is recognized by the endosomal sorting complex required for transport (ESCRT) containing Hepatocyte growth factor-regulated tyrosine kinase substrate (HGS, also known as Hrs) and Tumor susceptibility gene 101 (TSG101) in the endosome and is then sorted to the lysosome for degradation [4,40]. The proteasome also is involved in the degradation of CFTR mutants from the PM, but the mechanism of how these different degradation pathways are selected remains unknown [43,44].

**Figure 3:**
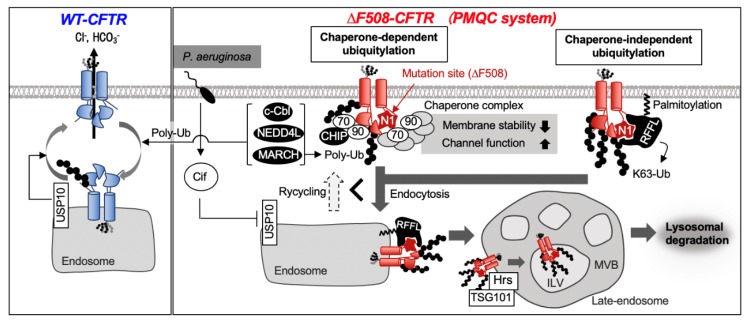
Wild-type (WT)– cystic fibrosis transmembrane conductance regulator (CFTR) exerts a stable channel function on the plasma membrane (PM). Internalized WT–CFTR could undergo deubiquitylation by ubiquitin Specific Protease 10 (USP10) and, consequently, be recycled to the PM. When *P. aeruginosa* infection occurs, USP10 is inhibited by CFTR inhibitory factor (Cif) produced by the bacteria, and CFTR turnover is accelerated. The misfolded region of ∆F508–CFTR is recognized by a chaperone complex at the PM. The chaperone complex helps to maintain the channel function of ∆F508–CFTR at the PM but promotes the CFTR elimination from the PM by facilitating the C-terminal Hsp-interacting protein (CHIP)-mediated ubiquitylation. Rififylin (RFFL) is localized at the PM and endosome and promotes endocytosis of ∆F508–CFTR by directly recognizing misfolded-nucleotide binding domain 1 (NBD1) and facilitating K63-linked poly-ubiquitylation. The endocytosed ΔF508–CFTR is invaded into the intraluminal vesicle (ILV) of the multivesicular body (MVB) by endosomal sorting complexes required for transport (ESCRT) proteins such as hepatocyte growth factor-regulated tyrosine kinase substrate (HGS, also known as Hrs) and tumor susceptibility gene 101 (TSG101) and sent to lysosomal degradation.

Ubiquitylation of ∆F508–CFTR at the PM involves the chaperone-dependent ubiquitin ligase C-terminal Hsp-interacting protein (CHIP) (Figure 3). ∆F508–CFTR on the PM is partially denatured, and its structural abnormality is recognized by chaperone complexes including Hsc70, Hsp90, and co-chaperones (DNAJA1, HOP, Aha1) [4]. The chaperone complex is vital for the maintenance of cell surface ∆F508–CFTR structural stability and channel function [45]. However, the unfolded cell surface of ∆F508–CFTR continues to bind to the chaperone complex similarly at the ERQC mechanism, and the chaperone-mediated CHIP binding promotes ∆F508–CFTR ubiquitylation [4,46]. It recently has been shown that the ubiquitylation of the unfolded cell surface of CFTR involves the chaperone-independent ubiquitin ligase rififylin (RFFL) (Figure 3) [47]. RFFL has a Fab-1, YGL023, Vps27, and EEA1 (FYVE)-like domain that binds to phosphatidylinositol (PI) 3-phosphate (PI(3)P) and PI(5)P in the N-terminal region, the Really Interesting New Gene (RING) domain which is important for ubiquitin ligase activity in the C-terminal region, and multiple disordered regions within other regions [48]. RFFL does not have a transmembrane domain, but its N-terminal domain is palmitoylated and is localized on the cytoplasmic side of the plasma and endosomal membranes [49,50]. RFFL directly recognizes the NBD1 of CFTR through the N-terminal disordered regions. Interestingly, the recognition by RFFL is dependent on the CFTR conformation, selectively recognizing and ubiquitylating partially denatured NBD1. RFFL knockdown has little effect on the ubiquitylation and degradation of immature ∆F508–CFTR localized at the ER. However, it suppresses the lysosomal degradation of mature ∆F508–CFTR from the PM by dramatically inhibiting the K63-linked polyubiquitylation, which is preferentially involved in lysosomal sorting [47]. Therefore, unlike CHIP, RFFL is thought to function as a PMQC ubiquitin ligase that directly recognizes the structural abnormality of ∆F508–CFTR located at the PM and endosome and directs it to degradation. Added to CHIP and RFFL, the ubiquitin ligases membrane-associated RING-CH 2 (MARCH2), c–CBL, and Nedd4L have been reported to participate in the CFTR degradation at the PM and post-Golgi compartment. However, it remains unclear if these ubiquitin ligases selectively recognize the structural abnormalities of CFTR and play an essential role in the CFTR quality control (Figure 3) [51,52,53,54].

De-ubiquitylating enzyme (DUB) is likely to be involved in the CFTR PMQC mechanism similar to ERQC. Endosome-localized DUB, Ubiquitin Specific Protease 10 (USP10), suppresses ubiquitylation of WT–CFTR in early endosomes and promotes the recycling of CFTR to the PM [55]. *Pseudomonas aeruginosa* virulence factor Cif, found in patients with CF and chronic obstructive pulmonary disease (COPD), suppresses USP10 activity and promotes CFTR ubiquitylation and lysosomal degradation (Figure 3) [56]. USP10 may recognize the conformational state of CFTR in endosomes and regulate its ubiquitylation level, although the direct evidence remains lacking. DUBs that recognize the structural state of CFTR and selectively deubiquitylates native CFTR have not been identified yet.

## 5. Cystic Fibrosis Transmembrane Conductance Regulator (CFTR) Modulators

Thirty years have passed since cystic fibrosis transmembrane conductance regulator (CFTR) was identified as the cystic fibrosis (CF) causative gene in 1989, and the molecular mechanism of CFTR expression and dysfunction due to a genetic mutation is being clarified [57]. During the past seven years, CFTR modulators including Kalydeco^®^ (2012), Orkambi^®^ (2015), Symdeko^®^ (2018) and Trikafta^®^ (2019) have been clinically used to correct the root causes of CF. Kalydeco^®^, also known as ivacaftor and VX–770, is the first CFTR modulator clinically used and acts as a CFTR potentiator facilitating the channel opening of class III and IV mutants, including G551D–CFTR. Orkambi^®^ is the first CFTR modulator for CF carrying ∆F508–CFTR. It is a combination drug of Kalydeco^®^ and lumacaftor (VX–809), which is a CFTR corrector facilitating the endoplasmic reticulum (ER) export and plasma membrane (PM) expression of the CFTR protein. Symdeko^®^ is also a combination drug of Kalydeco^®^ and tezacaftor (VX–661), which has an improved drug interaction. The CFTR corrector lumacaftor and its analog tezacaftor stabilize the CFTR structure by improving the inter-domain interaction between membrane spanning domain (MSD)1,2 and nucleotide binding domain 1 (NBD1) in addition to the effect of improving the folding of MSD. However, lumacaftor and tezacaftor fail to improve the conformational stability of NBD1 itself [58,59,60]. Actually, both Orkambi^®^ and Symdeco^®^, which are combined drugs with ivacaftor, have limited effects in clinical use [61]. The recently developed elexacaftor (VX–445) increases the PM expression of ∆F508–CFTR when used in combination with the first-generation CFTR corrector (lumacaftor or tezacaftor) and, thus, may have a different point of action than the first-generation CFTR correctors [62]. Since the effectiveness of the first-generation corrector is synergistically enhanced by the NBD1 stabilization, elexacaftor may have an NBD1 stabilizing effect. Elexacaftor, tezacaftor, and ivacaftor were approved by the Food and Drug Administration (FDA) as Trikafta^®^. Trikafta^®^ improves the indicator of respiratory function (% forced expiratory volume in one second (FEV1)) by about 14% after administration for four weeks, while Symdeco^®^ improves about 7%, indicating a high clinical efficacy of Trikafta^®^ [62,63].

## 6. Cystic Fibrosis Transmembrane Conductance Regulator (CFTR) Ubiquitylation as a Cystic Fibrosis (CF) Drugs Target

Since the accelerated cystic fibrosis transmembrane conductance regulator (CFTR) degradation causes the loss of function of CFTR in cystic fibrosis (CF), attenuating the CFTR degradation could be useful for the treatment of CF. However, treatment with proteasome inhibitors suppressing the ∆F508–CFTR endoplasmic reticulum associate degradation (ERAD) fails to restore the CFTR function; instead, they accumulate ubiquitylated ∆F508–CFTR in the cytoplasm and form aggresomes [64]. Conversely, overexpression of the endoplasmic reticulum (ER) molecule chaperone calnexin (CNX) inhibits ∆F508–CFTR ubiquitylation and retro-translocation to the cytoplasm and, consequently, foldable ∆F508–CFTR that can be expressed at the plasma membrane (PM) is accumulated on the ER membrane [10]. Since ubiquitylation is thought to promote the retro-translocation of ∆F508–CFTR, counteracting the CFTR-selective ubiquitylation by regulating ubiquitin ligases could be useful for the treatment of CF [65]. Indeed, in cell line models, knockdown of ubiquitin ligases Ring finger protein 5 (RNF5), C-terminal Hsp-interacting protein (CHIP), or synoviolin 1(SYVN1), involved in ERAD of ∆F508–CFTR enhances the efficacy of the CFTR corrector [32,66]. Seen in mouse models, it has been shown that RNF5 knockout improves ∆F508–CFTR expression and function, resulting in ameliorated intestinal malfunction [67]. Additionally, an RNF5 small molecule inhibitor (Inh–02) improves ∆F508–CFTR expression and function in a primary airway epithelial cell of CF patients [68]. Therefore, CFTR-associated ubiquitin ligase inhibitors are useful as add-on drugs that enhance the limited efficacy of CF drugs, including Orkambi^®^. CHIP participates in the CFTR ubiquitylation at the ER and PM. However, counteracting CHIP activity may not be preferable for a CF therapeutic approach as its knockout has a deleterious effect [69]. Rififylin (RFFL), another ubiquitin ligase responsible for the CFTR plasma membrane quality control (PMQC), seems to be a promising target for CF treatment because RFFL knockout mice exhibit no abnormal phenotypes [47,70]. Since RFFL knockdown improves ∆F508–CFTR PM stability, RFFL inhibitors also are expected to be CFTR stabilizers, a first-in-class CF drug, which helps maintain functional ∆F508–CFTR at the PM and improves the limited efficacy of the CF drug Orkambi^®^ [71].

## 7. Conclusions

The majority of cystic fibrosis (CF) in patients is caused by abnormal cystic fibrosis transmembrane conductance regulator (CFTR) folding, thus, understanding of the CFTR protein quality control mechanism requires the identification of new CF therapeutic target molecules which can then contribute to the development of CFTR modulator enhancers based on new molecular mechanisms. Particularly, ubiquitylation of CFTR is complicatedly controlled by many regulatory factors at various locations in the cell, but its selective inhibition can be applied to the treatment of CF. Currently, ubiquitin ligases are focused on potential therapeutic targets for CF, but the number of small molecules that selectively inhibit the ubiquitin ligases is limited. Someday, the use of various modalities, such as nucleic acid medicines, peptide drugs that are effective in inhibiting protein-protein interaction, and degradation inducers such as PROTAC (proteolysis-targeting chimeric molecule), will lead to highly-satisfying CF drug therapy.
